# Analytical Characterization of Water-Soluble Constituents in Olive-Derived By-Products

**DOI:** 10.3390/foods10061299

**Published:** 2021-06-05

**Authors:** Pablo Doménech, Aleta Duque, Isabel Higueras, José Luis Fernández, Paloma Manzanares

**Affiliations:** Advanced Biofuels and Bioproducts Unit, Department of Energy—CIEMAT, Avda. Complutense 40, 28040 Madrid, Spain; pablo.domenech@ciemat.es (P.D.); isabel.higueras@ciemat.es (I.H.); jose.fernandez@ciemat.es (J.L.F.); p.manzanares@ciemat.es (P.M.)

**Keywords:** olive tree, water-soluble extractives, olive tree pruning, olive leaves, olive stones, extracted olive pomace

## Abstract

Olive trees constitute one of the largest agroindustries in the Mediterranean area, and their cultivation generates a diverse pool of biomass by-products such as olive tree pruning (OTP), olive leaves (OL), olive stone (OS), and extracted olive pomace (EOP). These lignocellulosic materials have varying compositions and potential utilization strategies within a biorefinery context. The aim of this work was to carry out an integral analysis of the aqueous extractives fraction of these biomasses. Several analytical methods were applied in order to fully characterize this fraction to varying extents: a mass closure of >80% was reached for EOP, >76% for OTP, >65% for OS, and >52% for OL. Among the compounds detected, xylooligosaccharides, mannitol, 3,4-dihydroxyphenylglycol, and hydroxytyrosol were noted as potential enhancers of the valorization of said by-products. The extraction of these compounds is expected to be more favorable for OTP, OL, and EOP, given their high extractives content, and is compatible with other utilization strategies such as the bioconversion of the lignocellulosic fraction into biofuels and bioproducts.

## 1. Introduction

Lignocellulosic biomass residues derived from agroindustries represent a valuable alternative source of material for the production of biofuels and bioproducts, which could replace those coming from conventional fossil sources. Among the different industries, the olive oil industry constitutes a large sector in Mediterranean countries, both culturally and economically: in Spain, a total of 2,733,620 ha were used in 2019 for the cultivation of olive trees, 60% of the total surface being concentrated in the southern region of Andalusia [1], for a total production of almost 6 Mt of olive biomass [2].

**Figure 1:**
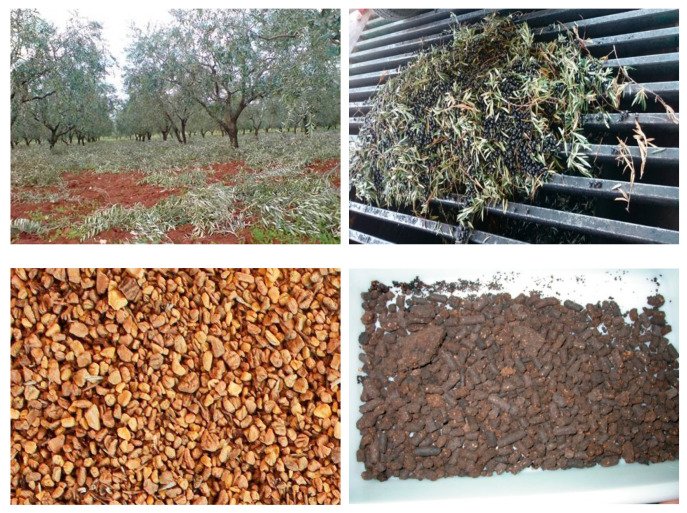
Olive by-products: olive tree pruning (top-left), olive leaves (top-right), olive stones (bottom-left) and extracted olive pomace (bottom-right).

This industry comprises several activities such as olive tree cultivation, olive collection, production of olive oil, and extraction of olive oil pomace. Besides these main products, the main solid by-products generated in the olive tree sector include olive tree pruning (OTP), olive leaves (OL), olive stones (OS), and extracted (or exhausted) dry olive pomace (EOP) [3]. Each of these by-products (see Figure 1) has unique characteristics, collection methods, and in the last years novel strategies for their valorization have emerged, making the term “residue” no longer suitable for them.

- OTP is the leftover biomass resulting from the pruning of olive trees during cultivation for an average production of 1.3 t/ha/y, and it is made up of approximately 30% leaves, 50% thin branches, and 20% olive wood [4]. It is a lignocellulosic material with a high content of carbohydrates (cellulose and hemicellulose, up to 55% of the total content) and thus holds great potential for use as a raw material within a biorefinery context for the production of biofuels and bioproducts. This by-product has attracted high research attention in recent years and has been investigated as a starting point for its conversion into bioethanol [5,6], cellulose nanofibers [7], xylitol, and antioxidants [4], or oligosaccharides with prebiotic potential [8], among many others;
- OL are originated in olive mills from cleaning operations of collected olives before entering the olive oil extraction process. They are typically disposed of through burning or used as animal feed; however, they are a great source of valuable organic substances like oleanoic acid, mannitol, and oleuropein [9]. The structural composition of OL is made up mostly of extractives (36%) and lignin (40%), with small contents of structural carbohydrates (6% cellulose and 4% hemicellulose) [10]. The high content of these extractable compounds in OL has focused the research on their extraction rather than on the use of OL as a substrate in biorefineries [11];
- OS are separated in olive mills or in olive pomace oil extracting industries before oil extraction and represent up to 15% of the total olive weight [9]. With a high calorific potential, OS is one of the most utilized residual biomasses for the self-generation of heat in agroindustries; up to 78% of all companies in Spain using solid biomass as fuel use OS [12]. Their structural composition consists of up to 50% sugar in the form of cellulose and hemicellulose and 25%–27% lignin [10]. This also grants them a potential use as a raw material in a biorefinery, as the research in this field focuses on its fractionation in order to maximize sugars liberation for further bioconversion [13];
- EOP is the leftover biomass obtained after the oil extraction of olive pomace, making up a 20% of the total dry mass of the pomace, which in turn constitutes 70%–80% of the total weight of the olive itself [14]. Pomace can be reused for further olive oil extraction by means of a solvent extraction process and a refining process and has been reported to be a source of high-value-added compounds such as phenols and polyphenols, vitamins, fatty acids, and other relevant antioxidants [10]. EOP is used as a solid biofuel for self-supply at small plants, though this application comes with high environmental impact due to the emission of hazardous particles and gases through combustion [14].

These four by-products have potential uses that could be framed within a multi-feedstock biorefinery based on the cultivation of olive trees for the production of different bio-based goods, such as biofuels (bioethanol) and other high-added value compounds as antioxidants and xylitol [9,15,16]. This approach can be considered as a step towards the transition to a sustainable bioeconomy in geographical zones with high disposal of lignocellulosic agricultural residues. In spite of the advances reached up to now, further research is still needed in order to make the most out of the full potential of each of them.

Any processing route aimed at successful fractionation and full valorization of a lignocellulosic biomass feedstock has to be founded on a sound knowledge of the composition and structure of the substrate. Among the different characterization methodologies, the ones described by Sluiter et al. [17,18] are commonly accepted as a reference for the complete compositional analysis of lignocellulosic biomasses. These analytical procedures measure the structural carbohydrates, lignin, extractable compounds, protein, and ash of the biomass. In the case of the four solid by-products from the olive oil sector above presented, the extractable fraction is particularly interesting from a biorefinery point of view since many high-value-added products can be found in it. While there are works detailing the presence and utility of several of them, knowledge of the full quantification of extractive compounds of olive biomasses is scarce, and to the best of our knowledge, there is no published work on a comparative quantification of these extractives within different by-products from the olive tree industry.

The removal and quantification of extractives in this kind of samples is part of the analytical procedure followed by Sluiter et al. [17,18] to fully characterize lignocellulosic biomass composition. According to this methodology, extractives can be either aqueous or organic, depending on whether they are obtained through an extraction process with water or with ethanol, respectively. Once these extracts are obtained and quantified, further research to reach a near-complete characterization can be carried out by applying a series of analytical procedures, such as those shown by Chen et al. for corn stover [19] and switchgrass [20] for a fraction of aqueous extracts. Following this research line, this work aims at fully characterizing the water extractives present in EOP, OTP, OL, and OS, including those that are typically overlooked. The final goal was to make an assessment of the presence of high-value-added compounds among them and a full comparison of the composition of water extractives between the four different olive biomasses, as well as their full composition in terms of structural carbohydrates and lignin, among others. 

## 2. Materials and Methods 

### 2.1. Olive By-Products Sample Preparation 

Extracted dry olive pomace (EOP) and olive stones (OS) were provided by local companies in Jaén (Andalusia, Spain), while olive tree pruning (OTP) and olive leaves (OL) were kindly supplied by the research group of the Chemical, Environmental and Materials Engineering at the University of Jaén. Each olive by-product sample was ground and then screened through a 40 mesh sieve and further stored for their compositional characterization. At the time of storage, the moistures for OTP, OL, OS, and EOP were 6.4%, 4.8%, 3.5%, and 6.2%, respectively.

### 2.2. Analytical Procedures for By-Products Chemical Composition and Extractives Quantification

The chemical characterization of all olive materials was carried according to the US National Renewable Energy Laboratory (Golden, CO, USA) analytical procedures for biomass analysis as defined by Sluiter et al. [17]. Briefly, the first analysis step consists of the quantification of the extractive content of the sample, which includes a sequential extraction process using water and ethanol. As the present work focused on the analysis of water extracts, the procedure followed for extractives quantification is shown in detail in the following section. Further analytical determination of carbohydrates and lignin was carried out in the extractives-free biomass sample, and the results refer to the whole sample, following the calculations described in the methodology proposed by the National Renewable Energy Laboratory (NREL) mentioned above.

The carbohydrate analysis was performed by a two-step acid hydrolysis process, after which the monomeric sugars released to the acidic solution are quantified by High-Performance Liquid Chromatography (HPLC). The content in glucans (in biomass samples analyzed corresponds to cellulose) and the polymers that make up the hemicellulose (xylan, arabinan, galactan, and mannan) was calculated based on the amount measured of their corresponding monomers, assuming that all glucose found in the acid hydrolysate comes from the cellulose and the remaining hexoses (galactose and mannose) and the pentoses (xylose and arabinose) were derived from hemicellulosic polymers. To convert the sugar content values into their corresponding polymers, the results for hexoses were divided by 1.1, while for pentoses, the values were divided by 1.12. These factors correspond to the loss of a water molecule occurring during acid hydrolysis of polymers into the monomeric sugars that are quantified by HPLC. Thus, the content in hemicellulose is the sum of the content of constituent polymers, calculated with the above-mentioned factors. In the same acid hydrolysate, the acetic acid content was also measured by HPLC to quantify the acetyl groups that are substituents of xylan chains. Lignin was also solubilized during the acid attack was determined as an “acid-soluble lignin” in the acid hydrolysate by spectrophotometry. 

The content of the lignin insoluble in acid (assimilate to Klason lignin) was quantified by weighting the solid residue remaining after the acid hydrolysis of the biomass sample. In parallel to carbohydrates and lignin analysis, the determination of ash content was carried out in a raw biomass sample by calcination in a muffle furnace at 575 °C.

All analyses were carried out in triplicate; their compositions are taken as the mean values along with their standard deviations.

For the water extraction step, samples of 5 g from each olive biomass were added to a Soxhlet cellulose thimble and extracted overnight with 250 mL of water as described by Sluiter et al. [18]. Heating was adjusted in order to achieve a siphon rate of approximately 2–3 cycles per hour. Samples of 2 mL from the aqueous extracts generated were transferred for their analytical characterization (Section 2.3), the remaining content was evaporated using a JP Selecta rotary evaporator, and the residues were then dried to constant weight in a vacuum oven at 40 °C. The amount of extractives in the sample, on a percent dry weight basis, was calculated as seen on the following equations, where ODW refers to oven dry weight:

(1) $$\%\;\mathrm{Extractives}=\frac{{\mathrm{Weight}}_{\mathrm{Flask}+\mathrm{Extractives}}{\;-\;\mathrm{Weight}}_{\mathrm{Flask}}\;}{{\mathrm{ODW}}_{\mathrm{Sample}}}\;\times \;100$$

(2) $$\mathrm{ODW}=\frac{\left({\mathrm{Weight}}_{\mathrm{Thimble}+\mathrm{Sample}}{\;-\;\mathrm{Weight}}_{\mathrm{Thimble}}\;\right)\;\times \;\%\;\mathrm{Total}\;\mathrm{Solids}}{100}$$

Once the aqueous extracts were collected, they were kept cold until they were analyzed for major constituents, as described below.

### 2.3. Compositional Analysis of Aqueous Extracts

The analytical methodology used for the identification and quantitation of water-soluble compounds in olive by-products required different types of chromatographic and spectrophotometric analyses. The procedures for sample preparation and clean-up associated with the different analytical methods developed during this study are described below, depending on the type of compound evaluated. 

#### 2.3.1. Sugars and Related Alditols

Monomeric sugars, sucrose, and related alditols were determined using a Waters Alliance HPLC system Model 2695 (Waters Corporation, Milford, MA, USA) and a Waters 2414 refractive index detector (RID). The column used was a CARBOsep CHO782 Carbohydrate and Biomass Analysis (300 × 7.8 mm) coupled with a CARBOSep-CHO-782/C guard column (Transgenomic, Omaha, NE, USA), operating at 70 °C with Milli-Q water (Millipore, MA, USA) as mobile phase (0.5 mL/min). Before being injected into the HPLC, samples were cleaned through a Microionex MB200 ion-exchange resin (Rohm and Haas, Spain) in order to remove compounds that could interfere with the sugar analysis. Analyte identification was based on retention time, and they were quantified using multipoint, external standard calibration curves.

To determine the amount of water-soluble oligomeric sugars in the aqueous extracts by HPLC, a prior mild acid hydrolysis with 4% (*w/w*) H_2_SO_4_ for 30 min at 120 °C was required. Once the samples had cooled to room temperature, the pH was adjusted to 5–6 with calcium carbonate and cleaned with the ion-exchange resin. These samples were transferred to autosampler vials and analyzed for total sugar content using the HPLC-RID method described above. The oligosaccharide content was thus determined by the difference between the monosaccharide amounts measured in aqueous extracts and the total sugar content measured in the corresponding hydrolysate.

#### 2.3.2. Carboxylic Acids

Organic acids were determined with a Waters HPLC equipped with a Waters Autosampler 2707 and 2414 RID. The ICSep COREGEL-87H3 (300 × 7.8 mm) column (Transgenomic, Omaha, NE, USA) was employed at 65 °C with a mobile phase of sulfuric acid 5 mM at a flowrate of 0.6 mL/min. Before being introduced into the chromatograph, 2 mL aliquots of aqueous extract were filtered through a 0.45 μm membrane or 0.22 μm if they showed greater turbidity. External standard calibration curves and retention time identification were also used in the determination of carboxylic acids.

#### 2.3.3. Inorganic Anions and Cations

Aqueous extract aliquots of 2 mL were analyzed by the Unit of Analytical Chemistry at CIEMAT using an ionic chromatograph DIONEX ICS-2000 (Thermo Fisher Scientific, Waltham, MA, USA), according to the method described by C. Garcia-Diego and M. Sanchez [21] for their content in inorganic anions (Cl^−^, NO^3−^, SO_4_^2−^, and PO_4_^3−^) and cations (K^+^, Ca^2+^, Na^+^, Mg^2+^, and NH^4+^). External standard calibration curves were employed in the determination of inorganic ions via ion chromatography.

#### 2.3.4. Determination of Total Phenolic Content

The total phenolic content, expressed as vanillin equivalents, was determined by means of a UV/Vis Biochrom Anthos Zenyth 200rt spectrophotometer according to the Folin-Ciocalteu (FC) colorimetric method [22,23]. Vanillin was used as a reference standard for plotting calibration curves. A volume of 0.02 mL of the water extract was diluted with 0.088 mL de-ionized water, and it was mixed with 0.0125 mL Folin-Ciocalteu reagent. The reaction mixture was incubated at room temperature for 5 min in the dark. Then it was neutralized with 0.08 mL sodium carbonate solution (7.5%, *w/v*). The reaction mixture was incubated at room temperature in the dark for 120 min, with intermittent shaking for color development. The absorbance of the resulting blue color was measured at 765 nm. The final content value was determined from the concentration of phenolic compounds in the aqueous extract obtained by the linear equation of a standard curve prepared with vanillin and expressed as mg/g vanillin equivalent of dry extract.

#### 2.3.5. Phenols by High-Performance Liquid Chromatography (HPLC)

To identify the phenolic compounds present in the samples, the extracts were analyzed using an Agilent 1100 series HPLC coupled to an Agilent 1200 series 1050 Photodiode-Array detector (DAD). This detector allows a total scan of wavelengths, obtaining the complete UV absorption spectrum for each chromatographic peak, thus performing the quantitative analysis of each compound at the wavelength of maximum absorption. The separation was carried out on an XBridge250 C18 (Waters, Milford, MA, USA) analytical column at 65 °C, protected with a pre-column XBridge BEH C18 5 µm (Waters, Milford, MA, USA). The mobile phase was: solvent A, buffer potassium phosphate pH 7, 10 mM, and solvent B, methanol. The separation was carried out using gradient elution following this elution program: 0–10 min 93% A and 7% B; 10–20 min, gradually changing to 80% A and 20% B; 20–35 min, 80% A and 20% B; 35–45 min gradually changing to original conditions 93% A and 7% B. The flow rate was 0.6 mL/min, the column temperature was set at 35 °C, and the injection volume was 10 µL. All samples were measured at 275 nm. Before being introduced into the chromatograph, the samples were filtered through 0.45 μm membranes or 0.22 μm if they showed greater turbidity.

#### 2.3.6. Proteins

The protein determination procedure required the measurement of sample nitrogen content. A Kjeldahl measurement was performed according to method 984.13 of the AOAC International [24], using a Tecator digestor and Foss Tecator Kjeltec 8200 Auto Distillation Unit, considering a nitrogen-protein conversion factor of 6.25 [15]. Approximately 0.5 g of each sample was hydrolyzed with 12.5 mL of concentrated sulfuric acid containing two copper catalyst tablets (Kjeltabs Cu-3.5, Foss Analytics, Hillerød, Denmark) in the heat block at 420 °C for 1 h. After cooling, 80 mL of water was added to the hydrolysates before neutralization with 50 mL NaOH 50% and titration with HCl 0.1 N.

## 3. Results and Discussion

### 3.1. Structural Composition of Olive By-Products

The results of the structural composition of the selected olive oil-derived biomasses were characterized according to the methods described in Section 2.1 (structural carbohydrates, lignin, ash, and acetate), Section 2.2 (extractives), and Section 2.3.6 (proteins), are shown in Table 1. 

As it is apparent from the results in Table 1, these four by-products from the olive oil sector had distinct compositions, depending on the part of the plant they come from and the level of processing they have experienced. Thus, OTP, containing a significant amount of thin branches and olive wood as well as a part of olive leaves, had a much higher structural carbohydrates content (about 35%) than OL alone, which only had 19% of the total dry weight. EOP, containing rests of the olive fruit, mainly, also presented a rather low content in cellulose (11%) and hemicellulose (12%). OS showed a considerable amount of cellulose and hemicellulose, close to 50%, which is the highest value among the four substrates. This relatively high carbohydrate content in OS and OTP could indicate a good potential for the utilization of these fractions as feedstocks to generate a sugar platform in a biorefinery context. Some examples of biorefineries based on OS that outline different valorization routes for the carbohydrates present in these biomasses were gathered in the review by Ruiz et al. [9]. Moreover, different strategies have been proposed to access the hexoses and pentoses from OS, such as acid hydrolysis followed by detoxification [25] or alkaline extrusion [13]. 

Regarding the other main component of lignocellulosic biomass, lignin, it constituted more than 20% of the total dry weight in all the four olive by-products, being OS the material with the highest lignin content, 36%. Within a biorefinery context, lignin can be used to improve the energy balance of the process through combustion and power production [4], but it can also be a source of antioxidants, as claimed by Gómez-Cruz et al. [26].

The inorganic fraction of the biomasses varied from less than 1% for OS to about 9% for OL and EOP. The way OL is collected means that this substrate may be contaminated with a certain amount of soil, explaining thus the higher ash content of this biomass compared with the others. 

Protein content was especially high in EOP (9.1%) and OL (8%), while OTP had more or less half the amount of proteins (3%) and OS had less than 1%. These values are similar to the ones determined for olive milled leaves and olive tree pruning by Gullón et al. [16], who found around 3% protein content in OTP and around 7% in OL. Protein content of around 3% was determined in OL by Heredia et al. [27], unlike the value reported in the present work. The protein content of the olive-derived by-products has been mainly studied in relation to the use of these substrates as feed resources for ruminants, and in this context, olive leaves and olive cake are the most common choices [28,29]. According to Rodríguez et al. [30], olive seeds, which make part of olive stones, have a high protein content, mainly olive seed storage proteins, similar to globulins in other plants. Baker et al. [31] reported that most of the protein contained in OL would be oleosins. Furthermore, Martín García et al. [28] characterized proteins in OL and found high levels of arginine, leucine, and valine, as well as low tyrosine and cysteine content, for total crude protein content of 7% to 13%, in agreement with the proteins estimated in the present work. 

According to the data shown in Table 1, the methodology applied was able to determine more than 95% of the constituents of the biomass on a dry weight basis for all the cases. However, the knowledge offered by the analytical procedures applied is limited regarding the extractives fraction, which is only gravimetrically quantified [20]. Extractives constitute a large fraction of three of the four biomasses selected. EOP presented the highest extractives content, which added up to 42%, followed by OL (35%) and OTP (28%), whereas the extractives content in OS was comparatively less important, with only 6%. The aqueous extractives fraction was higher in all the four olive by-products analyzed compared to the organic fraction. The difference was as big as 6–7 times in the case of OTP and EOP. Several valuable compounds have been identified in the past in the extractable fraction of OTP [16], EOP [26,32], and OL [33,34], and the valorization of this fraction has been proved to be essential, for instance, to achieve the economic feasibility of a biorefinery based on OTP [4]. All this highlights the interest of fully characterizing the aqueous extractives fraction and identify as many water-soluble components as possible, which is the object of the next part of the work.

### 3.2. Sugars and Alditols

Sugars and alditols constitute one of the main easily identifiable fractions in the water extracts of lignocellulosic materials [19,20]. In the present study, the aqueous extractives recovered from the four selected olive by-products were analyzed for the content in free-sugars, oligomeric sugars, and related alditols, and the results are shown in Table 2.

The sugar content in the aqueous extractives was very different among the four materials but was, in any case, a substantial fraction of the total weight of water-extractable compounds (between 20% and 41%), as shown in Table 2. Although OTP and EOP extractives are rich in monomeric sugars, mainly glucose and fructose, a significant part of the total sugars were found in an oligomeric form. Oligomers constituted around 50% of the total sugar content in OTP water extractives, 42% in the case of EOP, and this oligomeric fraction went up to 82% of the total sugars for OS. Furthermore, the sugar profile was also very different for OS compared to the other three by-products: glucose was the main sugar determined in OTP, OL, and EOP water extracts, whereas xylose was the predominant sugar in the case of OS. In this regard, xylooligosaccharides obtained from the pretreatment of OTP have been demonstrated to have prebiotic properties [8], and so it could be interesting to run a similar study on the oligosaccharides found in OS. However, the low amount of extractives with respect to the total OS weight means that the xylooligosaccharides production should be complemented with pretreatment of the material that would yield a higher sugar concentration. Otherwise, the solubilized sugars could be integrated with a sugar-platform biorefinery through their conversion into valuable bioproducts. 

Regarding alditols, the four samples were analyzed for the presence of glycerol, xylitol, arabinitol, sorbitol, and mannitol, being the latter the only compound present above the detection range as seen in Table 2. Mannitol was particularly abundant in OTP, constituting almost 14% of the total weight of extractives, whereas the presence of this alcohol in OS water extract was almost negligible (<0.5%). Mannitol is one of the main photosynthetic products and a transport sugar in olive leaves [34], and it is known to take part in the osmotic adjustment mechanism that olive trees adopt to face drought [35]. This strategy consists of the accumulation of mannitol (among other osmotically active compounds) in the plant cells, helping the olive trees to maintain cell turgor and leaf activities and to improve water extraction from the soil. In terms of potential uses, mannitol is a low-calorie sweetener widely used in the food industry and as an excipient in pharmaceutical formulations; it can be used to produce resins and surfactants, it has several applications in the medical field, and it has also been explored as biofuel precursor [36,37]. This alditol can be industrially obtained by chemical, fermentative, or enzymatic methods from fructose or mixtures of fructose and glucose, but it can also be extracted from natural sources since it can be found in a variety of plants and algae [36]. Recently, Lama-Muñoz et al. [34] studied the mannitol content in olive leaves from different cultivars and optimized the extraction technique, obtaining between 3% and 8% mannitol using the Soxhlet method depending on the origin of the leaves, a range that is in agreement with the value found in the present study.

### 3.3. Organic Acids

The results of the study of organic acids in the four olive-derived by-products are summarized in Table 3.

Acetic acid was detected in the four samples, as can be seen in Table 3. Along with acetic acid, another peak appeared in the chromatogram in the range of organic acids retention time, but it could not be identified clearly with a particular carboxylic acid. Thus, it was quantified as “other acids” in Table 3. Among the four olive by-products, OS was the one with the highest organic acid content, with as much as 16% of the total aqueous extractives weight, and this content was equally divided between acetic and other acids. In previous studies, several organic acids were detected in the water-soluble fraction of different lignocellulosic materials [19,20].

Acetic acid comes mainly from the hydrolysis of acetyl groups in hemicellulose [38]. Unlike what was reported for corn stover [19] or switchgrass [20], the significant presence of this kind of aliphatic acids in the aqueous extracts of the four selected olive by-products indicates that, during the extraction process, there is a certain limited autohydrolysis that affects mainly hemicellulose, since the amount of organic acids found is higher in the substrate with the highest hemicellulose content, OS (Table 1). Acetic and also other organic acids have been identified as inhibitory products regarding yeast fermentation when they are present in the medium over a certain concentration, although in small amounts, there is some evidence that they could be even beneficial for fermentation [39]. 

Recently, the potential of acetate as an alternative carbon source for the production of multiple fermentation chemicals has been noted [40]. The authors highlight the importance of an efficient (co-)utilization of acetate along with glucose or other sugars to increase the product yields, minimize the formation of by-products and promote the (co-)utilization of complex raw materials through microbial cell factories. Among others, acetic acid is the precursor in the chemical industry of vinyl acetate monomer, acetic anhydride, acetate esters, and purified terephthalic acid [41].

A significant concentration of acetic and other aliphatic acids in the water-soluble fraction of OS could indicate a good potential for the recovery of these products from hemicellulose in this substrate when submitted to adequate treatment.

### 3.4. Inorganic Compounds

Aqueous extracts of the different olive by-products showed varying concentrations of inorganic compounds, most notably metallic cations in higher amounts. The breakdown of these values, distinguishing between cations and anions, is shown in Table 4.

The amount of inorganic compounds for all by-products was formed from mainly cations, with potassium being the most abundant one in all cases, reaching up to 5.36% for EOP. Calcium was the second most abundant cation for OL (0.70%) and EOP (0.34%), sodium for OTP (0.25%) and ammonium for OS (0.69%). Magnesium seemed to have a residual presence for OTP and OS, with values lower than 0.1%, the same as ammonium for OTP (not detected), OL, and EOP. On the other hand, the presence of anions was considerably lower, being less than 1% for all by-products. Sulphate was the most abundant for OTP (0.53%) and OL (0.81%) followed by chloride (0.08% and 0.13% respectively), while the opposite case occurs for OS (0.32% chloride and 0.06% sulphate) and EOP (0.52% chloride and 0.41% sulphate). Traces of nitrate (0.01%) were found in extractives from OTP and OS, while phosphate (PO_4_^3−^) was looked for but not detected in any of the four cases.

Literature addressing the presence of inorganic compounds in the form of ions is scarce for the olive by-products studied in the present work. In their similar work on the composition of extractives from switchgrass [20] and corn stover [19], Chen et al. noted considerably higher amounts in terms of inorganic ions: ranging from 7% to 13% for cations and 3%–5% for anions. Olive by-products share a similar tendency as in a higher proportion of cations. In fact, potassium (the most abundant cation found in all four cases) is commonly found in olive-derived products, such as olive oil mill wastewaters [42] or olive pomace [43]. Chloride is typically detected as the most abundant anion in virgin olive oil, followed by sulfate [44], in line with the results herein obtained.

### 3.5. Phenolic Compounds

The content of phenolic compounds in aqueous extracts was analyzed according to two different methods as explained in Section 2.3.4 (FC) and Section 2.3.5 (HPLC), the former determining the total content of all phenolic compounds and the latter for the specific quantification of certain products. The results are summarized in Table 5.

In all four olive by-products, the presence of phenolic alcohols was considerably higher than that of acids. The most abundant phenolic compound detected for OTP and OL was 3,4-dihydroxyphenylglycol (DHPG), at 1.14% and 1.04%, respectively, which seemed to be non-existent for OS (tyrosol, Tyr, was the most abundant phenolic compound: 1.10%) and EOP (hydroxytyrosol, OH-Tyr, the most abundant: 1.10%). As per phenolic acids, vanillic acid was only detected on OS (0.21%), where syringaldehyde also showed a relevant quantity (0.10%). No phenolic acids were detected at all in OL and EOP, while only a small 0.01% of syringaldehyde was found for OTP. Syringic and ferulic acids were looked for but not detected in any of the aqueous extracts derived from the four olive by-products.

Several of the compounds detected are known to have potential uses. OH-Tyr and DHPG are two antioxidant compounds that are commonly found in olive-derived biomass or olive oil itself. OH-Tyr has been largely addressed as a potent antioxidant derived from olives [45] or wine [46], while DHPG was studied by Lama-Muñoz et al. [47] along with OH-Tyr and α-tocopherol as enhancers of the stability of different vegetable oils against oxidation. These two compounds are commonly studied together in a plethora of applications within the food sector: preservation of meat [48], dietary supplement promoting intestinal health [49] or quality enhancers of ovine semen [50]; and are known to be important contributors to the anti-inflammatory properties of virgin olive oil [51]. Vanillin, the phenolic alcohol found in the lowest amounts in the present study, also presents several applications of interest, most notably as a sweetener in the food industry. However, synthetic production of vanillin is widely established, either using oil (85%) or lignin (15%) as raw material [52]; thus, the suitability of vanillin extraction from olive by-products is questionable given the low amounts detected. Vanillic acid is the oxidized form of vanillin and an intermediate in its synthesis, though it has found some uses in the food industry. 

The total phenolic content, as determined using the FC methodology, was considerably higher for all four olive biomasses than the specific compounds detected by HPLC. Other phenolic compounds that are commonly detected in these by-products include oleuropein apigenin-7-O-glucoside, luteolin-7-O-glucoside, quercetin, and verbascoside in OL or EOP [32,34]. Oleuropein and the diadehydic form of decarboxymethyl oleuropein (3,4 DHFEA-EDA) have also been reported in OS [53].

### 3.6. Overall Extractives Composition

**Figure 2:**
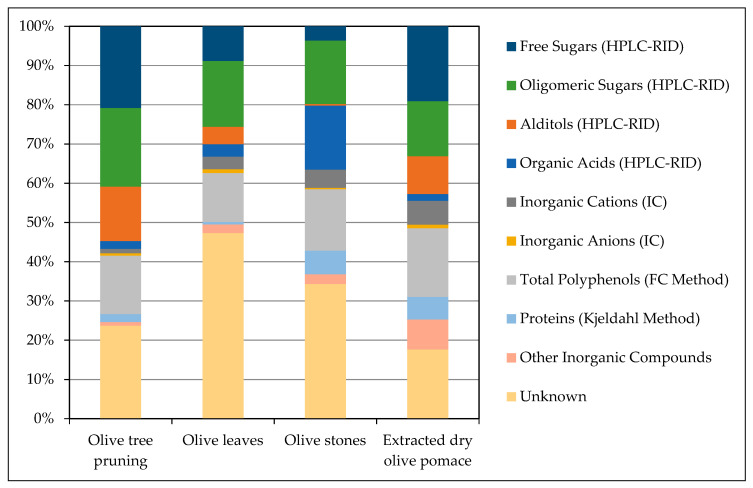
Overall composition of aqueous extractives for each olive by-product. HPLC-RID: High performance liquid chromatography-refractive index detector; IC: Ionic chromatography; FC: Folin-Ciocalteu.

In this section, a final roundup and mass balance of the composition of the aqueous extractives by raw material was carried out. Figure 2 shows the contribution of each of the categories of detected compounds to the total extractives weight.

The predominance of the different classes of components determined varies through each of the aqueous extracts of the four olive-derived by-products. Free sugars were the highest constituents of OTP (21%) and EOP (19%), while oligomeric sugars were of OL (16%) and a close-to-highest of OTP (20%) and OS (16%). Overall, sugars are the main identified compounds in the water-soluble fraction of the four biomasses selected.

The presence of mannitol, the only alditol detected in the samples, was particularly significant in the case of OTP (14%) and EOP (10%), while it was almost negligible for OS (<0.5%).

In contrast, organic acids were predominantly found in OS (16%), being considerably smaller contributors to the total extractives for the remaining by-products analyzed. As it was previously discussed, this fact could be related to the susceptibility of OS to the hydrolysis of hemicelluloses in the conditions of Soxhlet extraction.

There is a certain amount of inorganic matter contained in the feedstocks that were solubilized during extraction and that were not identified as inorganic cation or anions; this group was labeled as “Other Inorganic Compounds” in Figure 2. The contribution of this unspecific fraction to the total weight of extractives was less than 1% for OTP, but the significance was greater for OL and OS (around 2%) and especially for EOP, where the amount of undetermined inorganic matter added up to almost 8% of the total extractives weight. Altogether, the inorganic fraction could suppose as much as 15% in the case of EOP, maybe related to the industrial processes accumulated that result in this residue.

Proteins are found in greater amounts in water extractives of EOP and OS, both around 6%, than in OTP (2%) or OL (0.6%). This latter value is particularly remarkable since the extracted OL characterized in Table 1 revealed high protein content (8%). Proteins have been identified as interesting high-value-added compounds in olive-derived residues, and studies have been performed to propose advanced extraction methods that maximize the recovery while preserving the functionality [54,55].

The content of total polyphenols, a very interesting fraction for its antioxidant properties, was in the same range for the four biomasses considered, between 12% and 17%.

With the results obtained in the present work, the analytical characterization of the components of aqueous extracts of different olive by-products resulted in different degrees of mass closure: 82% for EOP, 76% for OTP, 66% for OS, and 53% for OL. This gives plenty of room for additional component characterization that was not fully addressed in this study while also considering the possibility of an insufficient determination with the methods provided. For instance, the FC-method used for phenol quantification may not have detected several bigger phenolic molecules that have been previously identified in these olive by-products [9], thus impeding a proper closure in these regards.

The lack of closure for mass balances is especially noteworthy for OL, where up to 47% of the total amount of extractives has not been characterized. OL is known to contain several non-structural components such as lipids, terpenes, carotenoids, and flavonoids [56] that may not have been detected in the analyses hereby carried out, while others might be present in the organic-soluble extractives fraction. Other compounds common to all biomasses that have not been determined in the present study and that could contribute to complete the characterization of the four biomasses are, for instance, fats, pigments, and sterols. Full mass closure for the extractive fraction of biomass is a complex matter given the huge amount of compounds that may be present in them, being common to end up with unknown fractions higher than 20% [19,20]. Therefore, there is still a need for further analysis of the aqueous fraction to be able to close the mass balances to an even greater extent. 

To complete the discussion, Table S1 shows the relative amount of each of the categories previously described with respect to the whole raw biomass. A great effort was carried out to try to define as much as possible the water-soluble fraction in the four olive-derived by-products. However, when looking at the whole picture, the very low amount of extractives in OS virtually ruled out any attempt to valorize the compounds found therein. In this regard, Hernández et al. [25] analyzed the techno-economic feasibility of a multi-product biorefinery of OS, and in their proposed strategy, all the different targeted products derived from the glucose or xylose-rich streams were obtained after the acid pretreatment of the whole biomass. 

On the other hand, monomeric and oligomeric sugars found in the extractives of OTP and EOP would increase the carbon source available with readily soluble sugars by adding almost 10% weight to the carbohydrate content in OTP and 15% to EOP, which could make a difference and influence the decision of using the whole biomass with or without extraction in a biorefinery context based on the sugar-platform. Nevertheless, it has to be taken into account as well the relatively high protein and phenols content in EOP (3% and 8% of total dry EOP weight, respectively) and consider if, in this case, a previous specific extraction of those compounds, as suggested by Gómez-Cruz et al. [26], would be more profitable. The high market price of antioxidants could also lead to choosing the inclusion of a first extraction step necessary to achieve a profitable process, as claimed by Susmozas et al. [4]. In the case of OL, it seems to be a more or less general consensus about the importance of recovering the bioactive compounds they contain [16,32,34,57] and consider these steps in the suggested configuration for a biorefinery [11]. The same conclusion could be reached for EOP and OTP, which also show a significant content of phenolic compounds with potential application as antioxidants.

## 4. Conclusions

Throughout this work, the aqueous extractives fractions of different olive-derived by-products were characterized, leading to a more detailed knowledge of their composition. Many of the high-value-added compounds that are most widely noted were confirmed to be present in the aqueous fraction of the extractives. This paves the way for a sequential valorization of these biomasses that several researchers have studied previously: a first extraction step where these valuable compounds (e.g., DHPG, mannitol) are obtained, followed by a bioconversion step where the structural carbohydrates are transformed into biofuels and/or bioproducts. This strategy seems to be more suitable for OTP, OL, and EOP, being the total amount of extractives more than 20% of the whole biomass, while for OS (where the extractives only amount for 6%), it may not be as profitable.

The level of characterization varied within each of them: while an almost-complete characterization was reached for the extractives of EOP, OL barely had half of their content determined. Further analysis needs to be carried out in order to fully close the content of these fractions: for instance, the search for fats, pigments, or sterols is proposed in order to determine the unknown fraction that remains after this work. Greater knowledge of the raw material is crucial in order to make an exhaustive use out of it within a biorefinery context, which is essential as the first step towards more sustainable agriculture by understanding the whole potential of all resources.

## Figures and Tables

**Table 1:** Biomass composition of different olive by-products in dry weight basis (dwb) of the raw biomass.

Component	OTP (%dwb)	OL (%dwb)	OS (%dwb)	EOP (%dwb)
Total extractives	27.8 ± 0.0	35.0 ± 0.0	6.3 ± 0.5	42.0 ± 1.2
*Aqueous*	*23.7 ± 0.0*	*21.9 ± 0.2*	*3.9 ± 0.4*	*37.5 ± 1.5*
*Organic*	*4.1 ± 0.2*	*13.2 ± 0.6*	*2.4 ± 0.2*	*4.5 ± 0.4*
Cellulose	20.8 ± 0.9	9.7 ± 0.1	20.9 ± 0.22	10.9 ± 1.0
Hemicellulose	14.5 ± 0.4	8.4 ± 0.1	26.0 ± 0.1	11.7 ± 0.6
*Xylan*	*9.2 ± 0.4*	*3.3 ± 0.1*	*23.5 ± 0.1*	*9.4 ± 0.4*
*Galactan*	*1.9 ± 0.0*	*1.9 ± 0.0*	*1.2 ± 0.0*	*1.0 ± 0.1*
*Arabinan*	*2.6 ± 0.1*	*2.8 ± 0.0*	*1.2 ± 0.0*	*1.0 ± 0.0*
*Mannan*	*0.8 ± 0.1*	*0.4 ± 0.0*	*0.1 ± 0.0*	*0.3 ± 0.0*
Lignin	22.6 ± 0.5	25.7 ± 0.0	35.6 ± 0.6	23.1 ± 0.5
*Acid-insoluble*	*19.7 ± 0.5*	*23.2 ± 0.0*	*33.9 ± 0.4*	*21.5 ± 0.4*
*Acid-soluble*	*2.9 ± 0.1*	*2.5 ± 0.0*	*1.7 ± 0.1*	*1.6 ± 0.1*
Ash	2.7 ± 0.2	9.1 ± 0.1	0.6 ± 0.0	9.0 ± 0.4
Acetyl groups	3.3 ± 0.1	1.6 ± 0.0	5.9 ± 0.1	2.1 ± 0.0
Proteins	3.4 ± 0.2	7.8 ± 0.0	0.7 ± 0.0	9.1 ± 0.2

**Table 2:** Presence of water-extracted sugars and other related compounds for each olive by-product (% *w/w* of aqueous extractives).

Component in Aqueous Extractives	OTP (% *w/w*)	OL (% *w/w*)	OS (% *w/w*)	EOP (% *w/w*)
Sugars (monomers)	20.8	8.8	3.6	19.1
*Glucose*	*13.4 ± 0.8*	*5.5 ± 0.5*	*0.8 ± 0.2*	*10.4 ± 1.2*
*Xylose*	*0.5 ± 0.1*	*0.5 ± 0.2*	*0.8 ± 0.1*	*0.45 ± 0.0*
*Galactose*	*0.5 ± 0.2*	*0.5 ± 0.1*	*0.3 ± 0.1*	*1.0 ± 0.2*
*Arabinose*	*0.5 ± 0.1*	*0.6 ± 0.2*	*1.1 ± 0.2*	*0.5 ± 0.1*
*Mannose*	*0.4 ± 0.2*	*0.3 ± 0.1*	*0.1 ± 0.1*	*1.6 ± 0.1*
*Fructose*	*5.6 ± 0.5*	*1.4 ± 0.2*	*0.5 ± 0.0*	*5.2 ± 1.0*
Sugars (oligomers determined as)	20.1	16.8	16.2	14.1
*Glucose*	*15.6 ± 3.3*	*10.4 ± 1.5*	*1.3 ± 0.2*	*7.6 ± 1.8*
*Xylose*	*0.2 ± 0.1*	*0.8 ± 0.4*	*10.1 ± 1.8*	*0.2 ± 0.1*
*Galactose*	*1.8 ± 0.5*	*2.7 ± 0.7*	*1.9 ± 0.1*	*2.4 ± 0.4*
*Arabinose*	*2.4 ± 0.6*	*2.5 ± 0.6*	*2.6 ± 0.3*	*3.6 ± 0.6*
*Mannose*	*n.d.*	*0.3 ± 0.2*	*0.3 ± 0.2*	*0.3 ± 0.3*
Alditols				
*Mannitol*	*13.9 ± 0.4*	*4.4 ± 0.4*	*0.4 ± 0.2*	*9.6 ± 1.6*

n.d.—not detected.

**Table 3:** Presence of water-extracted organic acids for each olive by-product (% *w/w* of aqueous extractives).

Component in Aqueous Extractive	OTP (% *w/w*)	OL (% *w/w*)	OS (% *w/w*)	EOP (% *w/w*)
Organic acids	2.0	3.2	16.3	1.7
*Acetic acid*	*0.3 ± 0.1*	*1.0 ± 0.2*	*8.4 ± 0.5*	*1.0 ± 0.0*
*Other acids*	*1.6 ± 0.1*	*2.2 ± 0.7*	*7.9 ± 1.2*	*0.7 ± 0.0*

**Table 4:** Presence of water-extracted inorganic compounds for each olive by-product (% *w/w* of aqueous extractives).

Component in Aqueous Extractives	OTP (% *w/w*)	OL (% *w/w*)	OS (% *w/w*)	EOP (% *w/w*)
Total Cations	1.16	3.24	4.60	6.07
*K^+^*	*0.71 ± 0.02*	*1.58 ± 0.07*	*2.96 ± 0.09*	*5.36 ± 0.10*
*Ca^2+^*	*0.12 ± 0.01*	*0.70 ± 0.01*	*0.22 ± 0.01*	*0.34 ± 0.01*
*Na^+^*	*0.25 ± 0.01*	*0.49 ± 0.01*	*0.65 ± 0.01*	*0.16 ± 0.00*
*Mg^2+^*	*0.08 ± 0.00*	*0.46 ± 0.01*	*0.09 ± 0.00*	*0.16 ± 0.01*
*NH_4_^+^*	*n.d.*	*0.01 ± 0.00*	*0.69 ± 0.01*	*0.04 ± 0.00*
Total Anions	0.62	0.95	0.39	0.93
*Cl^−^*	*0.08 ± 0.01*	*0.13 ± 0.01*	*0.32 ± 0.01*	*0.52 ± 0.03*
*NO_3_^−^*	*0.01 ± 0.00*	*n.d.*	*0.01 ± 0.00*	*n.d.*
*SO_4_^2−^*	*0.53 ± 0.06*	*0.81 ± 0.02*	*0.06 ± 0.00*	*0.41 ± 0.02*
Other Inorganic Compounds	0.91	2.19	2.49	7.64

n.d.–Not detected.

**Table 5:** Presence of water-extracted phenolic compounds for each olive by-product (% *w/w* of aqueous extractives).

Component in Aqueous Extractives	OTP (% *w/w*)	OL (% *w/w*)	OS (% *w/w*)	EOP (% *w/w*)
Total Phenolic Alcohols	2.02	1.33	1.58	1.31
*Tyr*	*0.06 ± 0.01*	*n.d.*	*1.10 ± 0.10*	*0.21 ± 0.08*
*OH-Tyr*	*0.80 ± 0.11*	*0.28 ± 0.03*	*0.31 ± 0.01*	*1.10 ± 0.32*
*DHPG*	*1.14 ± 0.03*	*1.04 ± 0.07*	*n.d.*	*n.d.*
*Vanillin*	*0.01 ± 0.00*	*0.01 ± 0.00*	*0.17 ± 0.17*	*n.d.*
Total Phenolic Acids	0.01	n.d.	0.31	n.d.
*Vanillic acid*	*n.d.*	*n.d.*	*0.21 ± 0.01*	*n.d.*
*Syringaldehyde*	*0.01 ± 0.00*	*n.d.*	*0.10 ± 0.01*	*n.d.*
Other Phenols	0.71	0.83	2.70	1.93
Total Phenols (HPLC) *	2.74	2.17	4.59	3.24
Total Polyphenols (FC)	14.9 ± 0.4	12.5 ± 0.2	15.6 ± 1.6	17.5 ± 0.9

n.d.: Not detected; HPLC: High performance liquid chromatography; FC: Folin–Ciocalteu. *: determined as the sum of all phenolic compounds characterized through HPLC.

## Data Availability

The data presented in this study are available in the present article and in supplementary material Table S1.

## Supplementary Materials

The following are available online at https://www.mdpi.com/article/10.3390/foods10061299/s1, Table S1: Presence of each compound category detected in the aqueous extractives fraction with respect to the whole biomass (% *w/w* of total biomass).
