# Supplementary material for: Analytical Characterization of Water-Soluble Constituents in Olive-Derived By-Products
Source: Foods. 2021 Jun 5;10(6):1299. doi: 10.3390/foods10061299 (PMC8229305; doi:10.3390/foods10061299)
Supplement: Supplementary file 1 [file foods-10-01299-s001.zip › foods-1211588-supplementary.pdf]

Table S1. Presence of each compound category detected in the aqueous extractives fraction with respect to the whole biomass (% w/w of total biomass)

**Compound**	**OTP**	**OL**	**OS**	**EOP**
Free Sugars	4.94	1.94	0.14	8.45
Alditols	3.29	0.97	0.02	4.26
Inorganic Cations	0.27	0.71	0.18	2.69
Inorganic Anions	0.15	0.21	0.02	0.41
Organic Acids	0.47	0.69	0.64	0.77
Oligomeric Sugars	4.76	3.67	0.64	6.23
Proteins	0.46	0.14	0.31	2.56
Other Inorganic Compounds	0.22	0.48	0.10	3.38
Total Polyphenols	3.54	2.73	0.62	7.75
